# Report from the 24th Annual Western Canadian Gastrointestinal Cancer Consensus Conference on Colorectal Cancer, Richmond, British Columbia, 28–29, October 2022

**DOI:** 10.3390/curroncol30090579

**Published:** 2023-08-29

**Authors:** Sharlene Gill, Shahid Ahmed, Brady Anderson, Scott Berry, Howard Lim, Terry Phang, Ankur Sharma, Joao Paulo Solar Vasconcelos, Karamjit Gill, Mussawar Iqbal, Keith Tankel, Theresa Chan, Magdalena Recsky, Jennifer Nuk, James Paul, Shazia Mahmood, Karen Mulder

**Affiliations:** 1 British Columbia Cancer Agency, Vancouver, BC V5Z 4E6, Canada; hlim@bccancer.bc.ca (H.L.); joao.solarvasconcelos@bccancer.bc.ca (J.P.S.V.); kgill@bccancer.bc.ca (K.G.); 2 Saskatchewan Cancer Agency, Saskatoon, SK S4W 0G3, Canada; shahid.ahmed@saskcancer.ca; 3 Western Manitoba Cancer Center, Brandon, MB R7A 5M8, Canada; banderson6@manitoba-physicians.ca; 4 Department of Oncology, Queen’s University, Kingston, ON K7L 3N6, Canada; scott.berry@kingstonhsc.ca; 5 Department of Surgery, University of British Columbia, Vancouver, BC V6T 1Z4, Canada; tphang@providencehealth.bc.ca; 6 Central Alberta Cancer Centre, School of Medicine, University of Calgary Cumming, Red Deer, AB T4N 6R2, Canada; ankur.sharma@albertahealthservices.ca; 7 Allan Blair Cancer Centre, Regina, SK S4T 7T1, Canada; mussawar.iqbal@saskcancer.ca; 8 Cross Cancer Institute, Edmonton, AB T6G 1Z2, Canada; keith.tankel@albertahealthservices.ca (K.T.); shazia.mahmood@saskcancer.ca (S.M.); karen.mulder@albertahealthservices.ca (K.M.); 9 British Columbia Cancer Agency, Surrey, BC V3V 1Z2, Canada; tchan4@bccancer.bc.ca; 10 Kelowna General Hospital, Kelowna, BC V1Y 1T2, Canada; magdalena.recsky@interiorhealth.ca; 11 British Columbia Cancer Hereditary Cancer Program, Victoria, BC V8R 6V5, Canada; jnuk@bccancer.bc.ca; 12 CancerCare Manitoba, University of Manitoba, Winnipeg, MB R3E 0V9, Canada; jpaul@cancercare.mb.ca

**Keywords:** adjuvant chemotherapy, colon cancer, colorectal cancer, immunotherapy, molecular tests, rectal cancer, surgery

## Abstract

The 24th annual Western Canadian Gastrointestinal Cancer Consensus Conference (WCGCCC) was held in Richmond, British Columbia, on 28–29 October 2022. The WCGCCC is an interactive multidisciplinary conference attended by healthcare professionals from across Western Canada (British Columbia, Alberta, Saskatchewan, and Manitoba) who are involved in the care of patients with gastrointestinal cancer. Surgical, medical, and radiation oncologists; pathologists; radiologists; and allied health care professionals such as dieticians, nurses and a genetic counsellor participated in presentation and discussion sessions for the purpose of developing the recommendations presented here. This consensus statement addresses current issues in the management of colorectal cancer.

## 1. Terms of Reference

### 1.1. Purpose

The aim of the Western Canadian Gastrointestinal Cancer Consensus Conference (WCGCCC) is to develop the consensus opinion of oncologists and allied health professionals from across Western Canada, attempting to define best care practices and to improve care and outcomes for patients with gastrointestinal cancers.

### 1.2. Participants

The WCGCCC welcomes medical oncologists, radiation oncologists, surgical oncologists, pathologists, radiologists, gastroenterologists, and allied health professionals from Western Canada who are involved in the care of patients with gastrointestinal malignancies (Table 1). Participants are provided the clinical questions to be addressed during the consensus conference in advance (Table 2).

### 1.3. Target Audience

The recommendations presented here are written for healthcare professionals involved in the care of patients with colorectal cancer (CRC).

### 1.4. Basis of Recommendations

The recommendations are based on presentation and discussion of the best available evidence. Where applicable, references are cited.

## 2. Question 1

What is the role of immunotherapy in deficient mismatch repair (dMMR) in early-stage rectal cancer? Can a non-operative approach be considered in patients who experience a complete response?

### 2.1. Recommendations

- All patients with rectal cancer should be tested for tumour mismatch repair (MMR) status.
- Compelling evidence supports a high clinical and pathologic response to immunotherapy for dMMR non-metastatic rectal cancer and can spare the need for chemotherapy, radiation, and surgery. Survival outcomes are not yet available.
- Ideally, immunotherapy should be considered as part of a clinical trial.
- Outside of a clinical trial (if not available), immunotherapy is reasonable to consider after a careful discussion with the patient regarding risks and benefits. These cases should be discussed within a multi-disciplinary team.
- The optimal immunotherapy agent(s) and duration of treatment are not known.
- In patients who achieve a complete clinical response, nonoperative management may be an option for those who are at high-operative risk or who decline surgery. These patients should be followed with intense surveillance per a watch-and-wait approach.

### 2.2. Summary of Evidence

Mismatch repair deficiency (dMMR) has been recognised as an important biomarker across numerous tumour sites that predicts sensitivity to immune checkpoint inhibition as a therapeutic strategy. The majority of dMMR cases are associated with Lynch syndrome and germline loss of function mutations in most commonly *MSH2* and *MSH6*. Hypermethylation of the *MLH1* promotor and *PMS2* loss of function mutations also incur the phenotype; however, they are less likely to be associated with germline mutations. The presence of coexisting *BRAF V600E* mutation strongly suggests a sporadic loss of MMR and not the inheritable form. Over the last few years, an increasing number of clinical trials evaluating the activity of immune checkpoint inhibitors including PD-1/PD-L1 (programmed cell death protein-1/programmed cell death ligand-1) and CTLA4 inhibitors in locally advanced colorectal cancer have shown promising responses and have even begged the question as to whether surgery can even be avoided in some dMMR diseases. The incidence of dMMR/microsatellite instability (MSI) is lower in rectal cancer compared to colon cancer (5.7% vs. 19.7%) [1,2].

Neoadjuvant chemotherapy alone is less likely to produce a meaningful response in dMMR colorectal cancer as compared to MMR proficient disease, and there is a higher risk of progression on therapy. When fluoropyrimidine is given concurrently with radiotherapy, responses appear to be similar to MMR proficient disease [3]. Combination chemotherapy with oxaliplatin does seem to maintain a survival benefit in the adjuvant setting [4].

A number of phase 2 and more recently some phase 3 trials have demonstrated significant activity of PD-1 inhibition, and in some cases in combination with CTLA4 inhibition for dMMR colon and rectal cancers. The KEYNOTE 177 trial has been the largest phase 3 trial conducted to date and has established pembrolizumab as first-line therapy superior to chemotherapy in MMR-deficient metastatic colorectal cancer [5,6,7,8,9].

The NICHE trial included 20 patients with dMMR locally advanced colorectal cancer who each received a single dose of ipilimumab and two doses of nivolumab prior to surgery. All 20 patients proceeded to surgery with 19/20 (95%) showing a major pathologic response defined as ≤10% tumour viability. Of these, 12/20 (60%) had a pathological complete response (pCR) at the time of surgery [10]. The PICC trial was a single-center parallel group randomised non-comparative phase 2 trial consisting of 2 groups of 17 patients harboring dMMR locally advanced colorectal cancer. Of these, 15/17 (88%) in the toripalimab + celecoxib group, and 11/17 (65%) of the toripalimab monotherapy group had a pCR at the time of surgery [11].

In 2022, the results of two key clinical trials suggest that immune checkpoint inhibition is likely poised to become the most integral component of treatment in dMMR locally advanced rectal cancers (LARC) [12,13]. First, Cercek et al. presented data at the American Society of Clinical Oncology (ASCO) 2022 Annual Meeting from a phase 2 single-arm trial of the PD-1 inhibitor dostarlimab. Twelve evaluable patients with Stage II or III disease received dostarlimab on a 3-weekly cycle for 6 months, to be followed by chemoradiotherapy and surgery for non-complete clinical response [12]. Of these patients, 12/12 (100%) experienced complete clinical response with no evidence of residual disease on magnetic resonance imaging, ^18^F-fluorodeoxyglucose positron emission tomography, endoscopic evaluation, digital rectal examination, or biopsy. At the time of publication, there were no grade 3 adverse events (AEs) reported (median follow-up of 6–25 months). No recurrences had been reported and no patients had gone on to receive chemoradiotherapy or surgery [12]. The NICHE-II trial presented at the European Society for Medical Oncology (ESMO) Congress 2022 was a colon cancer and not a rectal cancer trial; however, given the significant similarity in disease characteristics, it is nonetheless very encouraging and provides far larger numbers than the Cercek et al. trial with dostarlimab in dMMR rectal adenocarcinoma. The NICHE-II trial enrolled 112 patients with cT3+ and/or N+ dMMR disease in the ITT population that received 3 mg/kg of nivolumab plus 1 mg/kg of ipilimumab in the first cycle, then only nivolumab in the second cycle 2 weeks later, followed by surgery within 6 weeks of enrolling [13]. Pathologic responses were defined as <50% residual viable tumour (RVT), with major pathologic response (MPR) defined as <10% RVT, including those with pCR in the tumour and <10% viable cells in positive lymph nodes. pCR was defined as 0% viability in both tumour and lymph nodes. Only 2/112 (<2%) patients had immune-related events delaying surgery by >2 weeks. Of 112 participants, 74% had radiographic stage III disease. Of 107 evaluable patients, 99% had a pathologic response, and 95% MPR rate. The pCR rate was 67%, and grade 3 or greater immune-related adverse events (irAE) occurred in only 4 patients, 2 of which were asymptomatic elevations in amylase/lipase [13].

The Cercek et al. trial with dostarlimab found that complete responses were not seen before 3 months. This would contrast with the data presented from the NICHE-II trial, which demonstrated that 2 doses of immune checkpoint inhibitor and surgery within 6 weeks of first treatment showed a tremendous rate of pCR. It is possible that combination immunotherapy with the addition of a single dose of CTLA4 led to faster responses than dostarlimab monotherapy; however, it is impossible to say comparing the two trials. Further data are needed to support the optimal approach including mono or combination immune checkpoint therapy, as well as duration prior to definitive resection. More data is needed to determine whether resection can be safely omitted in those with complete clinical responses, with long-term outcomes comparable to other current standard therapy.

It has been an increasingly common question in the proficient mismatch repair (pMMR) rectal cancer cohort, with the recent evolution of data supporting, that some patients can be spared surgery with reasonable outcomes; however, long-term confirmation of this approach is still awaited and patient selection is critical to this approach. With the phenomenal and deep responses seen with neoadjuvant checkpoint inhibition in the dMMR cohort, one can only believe a similar watch-and-wait strategy used in the Organ Preservation in Rectal Adenocarcinoma (OPRA) trial could be employed following neoadjuvant immunotherapy in LARC, for patients experiencing a complete clinical response. The Cercek et al. trial has had reassuring findings omitting traditional chemoradiation and surgery thus far for all 12 patients that had clinical complete response to 6 months of dostarlimab. This approach is analogous to the OPRA trial which showed the superiority of chemoradiotherapy followed by neoadjuvant chemotherapy compared to reverse sequencing; however, the trial did not have a comparator arm of standard of care [14]. It did, however, establish reasonable outcomes with an organ preservation approach in rectal cancer, and thus we can glean from this that a similar strategy in the dMMR is not unreasonable.

## 3. Question 2

Can circulating tumour DNA (ctDNA) be used to tailor adjuvant chemotherapy in stage II colon cancer?

### 3.1. Recommendations

- The prognostic value of ctDNA in stage II colon cancer is well established, however, the predictive value of ctDNA is still under investigation and requires longer follow-up of currently available trials. Additional clinical trials are forthcoming, that will provide further direction as to the role of ctDNA in tailoring adjuvant chemotherapy in patients with stage II colon cancer.
- Patients should be offered clinical trials where available.

### 3.2. Summary of Evidence

Liquid biopsy can be described as the use of body fluids such as blood, liquor, saliva, effusions, urine, and stool as a source for tumour-derived molecular information [15]. Blood is the most studied specimen for liquid biopsy. There are a number of possible analytes that can be tested for on blood-based liquid biopsies such as circulating tumour cells (CTCs), tumour-educated platelets (TEPs), exosomes, circulating nucleic acids, proteins and metabolites [16].

Cancer and normal cells shed nucleic acids into the blood via secretion, apoptosis, or necrosis. Circulating tumour DNA (ctDNA) is the fraction of the total cell-free DNA (cfDNA) in the blood originating from cancer cells [17]. ctDNA is usually found as small fragments of nucleic acid of about 143–145 base pairs, with a half-life between 16–150 min [18,19,20]. Its relative abundance in the bloodstream ranges from less than 0.1% to more than 10% and can be referred to as variant allele frequency (VAF). Several factors can interfere with the presence of ctDNA in the blood, the most important being tumour location, cancer stage, disease burden, treatment, inflammation, infection, and trauma [18].

The majority of the data for clinical use of ctDNA comes from studies in the metastatic setting where assays have shown utility in providing comprehensive genomic profiling of cancer. Additional potential applications in the advanced disease scenario include monitoring therapeutic response, assessing treatment resistance and clonal evolution [21].

More recently, with progress in DNA sequencing technology, ctDNA use has been investigated in early-stage cancer for the detection of minimal residual disease (MRD) after curative-intent treatment. MRD is a concept more commonly used in hematology and relates to the detection of submicroscopic disease that cannot be assessed radiographically in a patient [21].

Detection of MRD is mostly a sensitivity game. Assays for MRD must be able to identify ctDNA at very low VAFs of 0.01%. Currently, this can be accomplished using polymerase chain reaction (PCR) and next-generation sequencing (NGS)-based methods [22].

Nevertheless, sensitivity can be affected by issues unrelated to assay characteristics. For instance, the timing of blood draws post-surgery may interfere with test results. An important work by Henriksen et al. showed that trauma from recent surgery can result in massive DNA shedding and ctDNA dilution to below the detection level resulting in a false negative report [23]. This is a good example to highlight the utmost importance of standardised procedures for pre-analytical and analytical steps in assays for the detection of MRD [22].

Finally, with the necessity of variant detection in such low levels, stoichiometry also plays a role as there is a chance that the mutation might not be detected in the blood sample because it is simply not there. Options to manage this are to collect more volume of blood, repeat the collection at different time points and look at additional mutations or epigenetic marks such as DNA methylation [24].

As tests are aiming for very high sensitivity, another important issue is specificity and the risk of false positives. The three major incidental findings for MRD detection that can interfere with specificity are germline mutations, sequencing errors, and clonal hematopoiesis of indeterminate potential (CHIP).

CHIPs are an age-related process where cells in the bone marrow accumulate mutations that can be detected in the cfDNA. Their frequency also increases with smoking and a history of anti-cancer treatment. This is particularly important because some of the mutations, such as *TP53* and *ATM*, can overlap with oncogenic driver mutations and present a significant challenge to differentiate between a CHIP and a cancer-related finding. Additionally, the finding of CHIP mutations in cfDNA may be a marker of increased risk of myelodysplastic syndrome, acute myeloid leukemia as well as cardiovascular disease [25,26].

There are different ways to deal with incidental findings. To reduce confusion due to potential sequencing errors, techniques such as the use of molecular barcoding and other unique molecular identifiers (UMIs) can be integrated into the bioinformatics pipeline. On the other hand, strategies to deal with CHIPs and germline mutations are sequencing of leukocytes in the buffy coat of the blood sample and the use of tumour-informed assays [22].

MRD assays can be grouped into two main categories. Tumour-informed platforms start with the profiling of a tissue sample to create a personalised targeted panel for a specific patient. This panel is then used to interrogate the peripheral blood for detection of ctDNA. As it requires prior information about the tumour, it tends to be costlier for the first test with lower costs for the subsequent ones, and with a longer turnaround time [27].

Tumour-uninformed platforms use predetermined NGS panels for frequent mutations and can be coupled with the analysis of methylation signatures to interrogate the peripheral blood for the detection of ctDNA. As they are agnostic panels, they do not require tissue samples, and tend to have a shorter turnaround time and lower cost [27].

Clinical validity is defined as the ability of an assay to divide, with statistical significance, one population into two or more groups based on outcomes [17]. For MRD detection using ctDNA technology, this has been extensively proven by several retrospective and prospective nonrandomised trials. Despite the high heterogeneity between those studies with respect to the patient population, disease stage, assay selection, testing schedule and follow-up, they are highly concordant in defining the detection of MRD after curative-intent treatment as one of the most significant prognostic factors for cancer recurrence. In some studies, the detection of ctDNA in this setting translates into a very high risk of relapse with impressive double-digit hazard ratios. Interestingly, the use of adjuvant chemotherapy is apparently able to clear about 16–67% of patients with positive ctDNA after surgery, and outcomes from some of those patients appear to be similar to the ones of patients with negative ctDNA after surgery [23,24,28,29,30,31,32,33,34,35,36]. Finally, data from the same studies shows that MRD presence can anticipate detectable recurrence in about 3 to 9 months when compared to standard clinical and imaging follow-up [23,24,28,29,30,31,32,33,34,35,36].

Clinical validity is very important, but a test is only useful if it can show clinical utility. This is defined as the ability to demonstrate—with statistical significance—improvement in the management of patients with the use of a particular test [17]. Well-designed and executed randomised controlled trials comparing the standard of care to new strategies utilising ctDNA for MRD detection are necessary for that. Several trials are ongoing in different populations of early-stage colorectal cancer but thus far, only the results of the DYNAMIC trial have been reported [37].

Adjuvant treatment for stage II colon cancer remains a clinical dilemma. Although most patients are cured by surgery, there are about 15–20% who will experience a recurrence [38]. The current standard of care is to consider the use of adjuvant chemotherapy in patients whose disease has clinicopathological characteristics of a higher risk of recurrence. Nevertheless, there is heterogeneity between guidelines regarding which patients should be considered for adjuvant treatment, and not all of the defined risk factors have similar prognostic significance [39,40,41]. Finally, data from randomised trials shows at best 5% absolute benefit in overall survival even in this highly selected population [42].

DYNAMIC is a phase 2 non-inferiority trial designed to investigate if the use of ctDNA to select patients with stage II colon cancer for adjuvant treatment could result in the reduction in chemotherapy use without compromising clinical outcomes. A total of 455 patients from 23 Australian centers underwent 2:1 randomisation into two groups after completing curative-intent surgery. In the ctDNA-guided management group, patients with detected MRD on week 4 or 7 after surgery received adjuvant chemotherapy, with the choice of regimen at the clinician’s discretion, whereas those with undetectable MRD underwent observation. In the control group, adjuvant treatment was decided based on the clinicopathologic criteria. High-risk was defined as proficient mismatch repair tumour (pMMR) with at least one additional of the following features: pT4, poorly differentiated, <12 lymph nodes yield, lymphovascular invasion, tumour perforation and/or bowel obstruction. The primary endpoint was two-year recurrence-free survival (2Y-RFS) [37].

The ctDNA test used for MRD detection in DYNAMIC was tumour-informed. Tumour tissue was analysed for mutations in 15 common genes in colorectal cancer such as *TP53*, *APC*, *KRAS*, *ATM* and *BRAF*. A personalised assay was built for each patient and subsequently used to interrogate plasma from blood samples collected sequentially on weeks 4 and 7 after surgery [37].

The study showed that the ctDNA strategy resulted in a statistically significant reduction in the use of adjuvant chemotherapy in comparison to the standard of care (15% vs. 28%; relative risk, 1.82; 95% confidence interval [CI], 1.25 to 2.65). In the ctDNA informed group, the time between surgery and initiation of adjuvant treatment was longer due to the turnaround for the test results. Finally, oxaliplatin-based adjuvant chemotherapy was more frequently used (62% vs. 10% *p* < 0.0001) in the ctDNA group [37].

After a median follow-up of 37 months, the 2Y-RFS was comparable between ctDNA-guided management and standard management (93.5% vs. 92.4%, HR 0.91; 95% CI, −4.1% to 6.2%). The absolute difference of 1.1% in favor of ctDNA and the lower limit of the 95% CI above the −8.5% threshold established by the study’s statistical design confirmed the non-inferiority of the ctDNA-based strategy [37].

Further analysis from the group of patients randomised to a ctDNA-based approach showed no statistically significant difference in 3-year recurrence-free survival (3Y-RFS), between untreated ctDNA negative patients and treated ctDNA positive ones (92.5% vs. 86.4%, HR 1.83; 95% CI, 0.79 to 4.27). Additionally, for untreated ctDNA negative cohort, pT4 tumours (HR 2.6; 95% CI, 1.01 to 6.71) and the presence of at least one high-risk clinicopathologic feature (HR 3.04; 95%; CI, 1.26 to 7.34) remained a significant prognostic factor [37].

More recently, a subsequent exploratory analysis from the ctDNA-guided cohort was presented at the ESMO Congress 2022 [43]. In this group, recurrence was seen in a total of 23 patients (7.9%). Postoperative ctDNA was negative in all 8 pts (100%) with locoregional relapse only, whereas 8 of 15 (53%) with distant relapse had a positive postoperative ctDNA (*p* = 0.02). The locoregional-only recurrence rate was greater in the ctDNA negative group compared to the ctDNA positive group (3.3% vs. 0%). Meanwhile, the distant recurrence rate was greater in the ctDNA positive group compared to the ctDNA negative group (13.6% vs. 3.4%). The clearance rate from post-operative ctDNA positive to negative was 87% and those patients had a far superior outcome (2Y-RFS of 97% vs. 1Y-RFS of 20%) than patients who did not clear MRD. The median time to recurrence for patients who remained ctDNA positive at the end of treatment was 5.3 months [43].

In summary, the DYNAMIC trial showed that ctDNA-guided therapy achieved comparable results to the current standard of care allowing less use of chemotherapy. The results seem particularly reassuring for omitting chemotherapy in ctDNA-negative patients with low-risk cancer. Additionally, the 87% MRD clearance rate observed is unprecedented and provides further favorable evidence of the utility of this strategy.

Nevertheless, there are several remaining questions before we can fully incorporate ctDNA in the management of stage II colon cancer patients. Firstly, ctDNA testing for MRD is not a perfect test and patients with negative results can still relapse, and this looks more significant for loco-regional relapses. Secondly, clinicopathologic high-risk features remained prognostically important in the group of ctDNA-negative patients. Therefore, a Bayesian approach seems more promising to increase the accuracy in patient selection for adjuvant treatment. Additionally, longer follow-up as well as larger sample sizes will be necessary to answer if we are increasing cure rates, or mainly delaying recurrence by clearing MRD with chemotherapy. Finally, the cost-effectiveness analysis is still pending.

In conclusion, the currently available data continues to support ctDNA as a very promising strategy for tailoring adjuvant chemotherapy in stage II colon cancer. Several ongoing trials will hopefully help to answer the remaining unanswered questions and allow us to fully understand how to incorporate this revolutionary technology into clinical care [27]. In the meantime, enrolling patients in those clinical trials is highly recommended.

## 4. Question 3

What is the role of chemotherapy in combination with monoclonal antibodies (mAbs) for the first-line treatment of patients with metastatic colorectal cancer?

### 4.1. Recommendations

- Patients should undergo timely molecular testing to determine an optimal first-line strategy; this would include MMR, *BRAF* and extended *RAS* analysis.
- In patients with pMMR, *RAS*/*BRAF* wild-type left-sided metastatic colorectal cancer, the recommended first-line treatment is combination chemotherapy where appropriate with an anti-epidermal growth factor receptor (EGFR) monoclonal antibody (mAb).
- In patients with pMMR, *RAS* or *BRAF* mutated or right-sided metastatic colorectal cancer (mCRC), the recommended first-line treatment is combination chemotherapy with bevacizumab.
- For right-sided mCRC, anti-EGFR mAB in the first line is not recommended.
- Patients should be offered clinical trials where available.

### 4.2. Summary of the Evidence

In 2017, Dr. Alan Venook presented data at the ASCO 2017 Annual Meeting that revealed that primary tumour location was an important, independent predictor of overall survival (OS) in patients with mCRC. These data were based on a retrospective analysis of North American Intergroup Trial CALGB/SWOG 80405/CCTG CRC.5, which compared first-line chemotherapy and cetuximab vs. chemotherapy and bevacizumab, in patients with *Ras* wild-type (WT) tumours [44]. These data demonstrated that patients with left-sided colon cancers had much better outcomes with anti-EGFR mAbs compared to patients with right-sided cancers. Subsequent retrospective analyses of other first-line trials of chemotherapy and anti-EGFR mAbs compared to chemotherapy and bevacizumab or chemotherapy alone showed similar results [45]. Two meta-analyses of the data confirmed these results. The Holch et al. meta-analysis focused on first-line trials, and demonstrated a benefit for overall survival (OS) for chemotherapy and anti-EGFR mABS in left-sided tumours vs. chemotherapy and bevacizumab (HR 0.71; 95% CI: 0.58–0.85; *p* = 0.0003), and (0.69; 95% CI: 0.58–0.83; *p* < 0.0001) a benefit for overall survival for chemotherapy and anti-EGFR mABs in left-sided tumours vs. chemotherapy alone [46]. For right-sided tumours, the hazard ratio for overall survival was 1.3 (HR 1.3; 95% CI: 0.97–1.74; *p* = 0.081). Although there were many limitations in these retrospective analyses, the consistency of the data across multiple trials, especially in the magnitude of benefit in OS in left-sided tumours (7–10 median OS benefit) was sufficient to change practice.

At the National Colorectal Cancer Sidedness Consensus Meeting in 2017, chemotherapy and an EGFR mAb were recommended as standard first-line treatment for patients with left-sided *Ras* WT tumours [45]. At the ASCO 2022 Annual Meeting, Yoshino and colleagues presented data from the prospective trial of FOLFOX and panitumumab vs. FOLFOX and bevacizumab as first-line treatment for patients with metastatic mCRC and left-sided *Ras* WT tumours in which the primary endpoint was OS in patients with left-sided tumours [47]. Panitumumab significantly improved median OS versus bevacizumab, in combination with FOLFOX (HR, 0.82; 95.798% CI, 0.68–0.99. *p* = 0.031) in left-sided tumours, affirming the results of the retrospective analyses and the choice of chemotherapy and an anti-EGFR mAB as the preferred first-line treatment for patients with mCRC and left-sided *Ras* WT tumours. Although the magnitude of benefit observed in this study was less than that observed in the retrospective studies, the hazard ratio for OS was similar.

## 5. Question 4

Which patients with newly diagnosed colorectal cancer should be referred for genetic screening?

### 5.1. Recommendations

- National guidelines with standardised criteria for hereditary cancer referral are needed.
- The following patients should be referred for genetic screening (Table 3):
  - Patients with dMMR colorectal cancer not attributed to *MLH1* promotor methylation.
  - Patients < 50 years of age at the time of diagnosis
  - Patients with a personal history of more than one Lynch syndrome-related tumour*
  - Patients with a first-degree relative < 50 years of age with a history of Lynch syndrome-related cancer*
  - Patients with 2 or more relatives with a history of colorectal or other Lynch syndrome-related cancer*
  - Patients with pathogenic or likely pathogenic variants found on tumour sequencing.

*Colorectal, endometrial, gastric, ovarian, pancreas, urothelial, brain (usually glioblastoma), biliary tract, small intestine, sebaceous adenomas, sebaceous carcinomas, and keratoacanthomas 

### 5.2. Summary of Evidence

The results of molecular genetic testing have become an integral part of planning treatment for patients with newly diagnosed colorectal cancer (CRC). The opportunity to provide targeted and personalised therapeutics is an additional advantage beyond the historic clinical value of genetic testing, which included a focus on cancer risk prediction for the patient and their biological relatives.

Recent studies have shown that using family history criteria alone to determine which patient with CRC should have germline genetic testing would miss a significant number of people with hereditary cancer syndromes [48]. Current guidelines, therefore, recommend a combination of personal pathology and/or family history.

**Figure 1:**
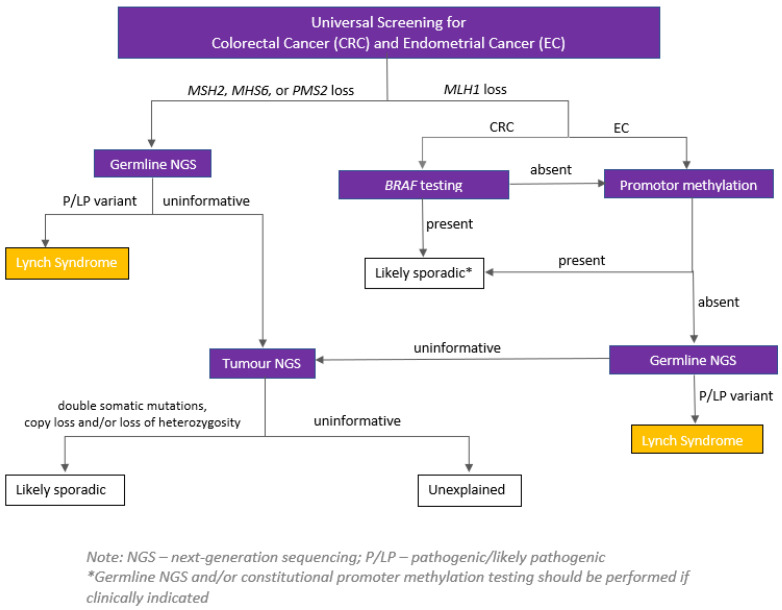
Example of a serial testing pathway for tumours identified as having dMMR through universal IHC. *Source*: Hereditary Cancer Program (2022), BC Cancer, Vancouver, British Columbia.

Universal screening of all newly diagnosed CRCs for deficient mismatch repair (dMMR) is recommended regardless of age [48,49,50,51]. This can be executed using either a microsatellite instability analysis (MSI) or immunohistochemistry (IHC), to detect the presence or absence of the MLH1, MSH2, MSH6 and PMS2 MMR proteins (with IHC being more widely available). Identifying dMMR serves two purposes: (a) recognising which patients may benefit from treatments such as immune therapy, and (b) identifying those who may have a diagnosis of the most common hereditary CRC syndrome—Lynch syndrome (Figure 1).

All patients with dMMR tumours where *BRAF V600E* and/or *MLH1* hypermethylation is not present should be referred for germline genetic testing. Of note, identifying *MLH1* hypermethylation lowers the likelihood of but does not rule out Lynch syndrome, as the hypermethylation may be the “second hit” in a person with a germline pathogenic variant in the *MLH1* gene, or in less common cases the person may have constitutional *MLH1* hypermethylation (i.e., this is present in all/most cells from birth).

As the presence or absence of dMMR cannot reliably diagnose or exclude Lynch syndrome in all patients, and given the phenotypic overlap of many hereditary CRC syndromes, offering multi-gene hereditary cancer panels to specific patient groups irrespective of MMR status is recommended [48,50,51]. With the decreasing cost of molecular genetic testing, this approach is becoming the standard of care to reduce the likelihood of a missed pathogenic variant.

Recent studies have shown a 16–18.3% detection rate for pathogenic variants in hereditary cancer genes for people diagnosed under age 50 with CRC [52,53]. While the majority of variants identified were in the MMR genes, biallelic *MUTYH*, *APC*, *SMAD4*, *TP53* and *CHEK2* variants were also reported. [52,53].

Yurgelun et al. reported a 22% (13/59) pathogenic variant detection rate in patients with CRC and at least one other Lynch syndrome-related primary cancer [54]. This lends support to and is consistent with the Revised Bethesda Guidelines which recommend genetic testing in any patient who has multiple Lynch syndrome cancers at any age (colorectal, endometrial, gastric, ovarian, pancreas, urothelial, brain (usually glioblastoma), biliary tract, small intestine, sebaceous carcinomas [48,50,51,55]. The degree of polyp burden, polyp pathology, and age at diagnosis all influence the likelihood of identifying a germline pathogenic variant in a patient with a newly diagnosed CRC in the context of polyposis. However, even patients with lower tubular adenoma counts (10–19) over time have a reported 5% or greater PV rate. Hamartomatous polyposis syndromes (e.g., *PTEN*-hamartoma tumour, Peutz–Jeghers, juvenile polyposis) and serrated polyposis syndrome may be diagnosed based on clinical diagnostic criteria either related to polyp pathology and burden alone, or on extra-colonic features in the patient and their family. Given phenotypic overlap and variable expression with germline variants in polyposis genes, germline genetic testing should be considered in patients with≥10 cumulative colorectal adenomas, ≥3 cumulative gastrointestinal hamartomatous polyps (hamartomas, ganglioneuromas, Peutz–Jeghers polyps, juvenile polyps) and in patients meeting World Health Organization criteria for serrated polyposis syndrome (SPS) (≥5 serrated lesions/polyps proximal to the rectum, all being ≥5 mm in size, with ≥2 being ≥10 mm in size) OR >20 serrated lesions/polyps of any size distributed throughout the large bowel, with ≥5 proximal to rectum [48,50,51,56]. The latter often have a mixed polyp phenotype that includes tubular adenomas. Genes implicated in a minority of these patients include *RNF43* and biallelic *MUTYH PV*.

Patients with a newly diagnosed CRC may already come from a family with a known pathogenic or likely pathogenic variant. Genetic testing in this context is helpful to explore the potential for the current patient’s CRC to be a phenocopy and to clarify the segregation of the variant in the family. Two recent studies have shown a high rate of pathogenic germline variants in patients with CRC and at least one first-degree relative with CRC. Current guidelines suggest relaxing previous age-specific criteria to include offering testing to all patients with CRC and a first-degree relative (FDR) with CRC or endometrial cancer at any age. In consideration of the spectrum of cancers associated with Lynch syndrome, a patient with CRC and one FDR or second-degree relative (SDR) with Lynch syndrome-related cancer diagnosed <50 y or ≥2 FDR or SDR with a Lynch syndrome-related cancer diagnosed regardless of age are recommended to have germline genetic testing [48,50,51].

Patients with a newly diagnosed CRC may have tumour sequencing to guide treatment decisions. Germline confirmation testing should be offered to all patients with positive results (i.e., pathogenic, or likely pathogenic variant identified in a relevant hereditary cancer syndrome gene). Patients who meet any of the above criteria and receive negative tumour sequencing results should be offered germline testing given that the two tests are often not equivalent in terms of genes tested, variant interpretation and scope of testing (e.g., tumour sequencing may not include analysis for copy number variants). Patients who have a germline pathogenic or likely pathogenic variant identified through private-pay testing, a clinical trial, or research should be referred to medical genetics to review the implications of the result for themselves and their families.

## 6. Question 5

Which patients with clinical stage II/III rectal cancer should preferably receive long-course chemoradiation over short-course radiation?

### 6.1. Recommendations

These cases should be discussed within a multi-disciplinary team.

- In a non-total neoadjuvant therapy (TNT) approach, long-course chemoradiation is recommended for T4 lesions, threatened mesorectal fascia (MRF), or those where the sphincter is threatened. In non-chemotherapy candidates, short-course radiotherapy (SCRT) followed by delayed surgery is also an option.
- For patients considered for a TNT approach, SCRT or long-course chemoradiation with neoadjuvant combination chemotherapy are reasonable options.
- For patients who cannot proceed or refuse radical-intent surgery, a non-operative approach can be considered [14].

### 6.2. Summary of Evidence

Some forms of neoadjuvant radiotherapy (RT) are considered to be the standard of care when treating locally advanced rectal cancer with curative intent. Classically, “long course” RT is given concurrently with either intravenous 5-fluorouracil (5FU), or oral capecitabine, and completed 6–8 weeks prior to total mesorectal excision (TME) with either low anterior resection (LAR) or abdominoperineal resection (APR). The RT dose is generally 45 Gray (Gy) in 25 fractions (fx) targets the pelvic lymph nodes with a rectal cone-down boost to 50–54 Gy in 3–5 fx to the gross tumour. A sequential or simultaneous integrated boost (SIB) technique can be used, and treatment planning is either 3-dimensional (3D) or utilises intensity-modulated radiotherapy (IMRT)/volumetric-modulated arc therapy (VMAT). Conventional long-course RT can be given in T3 or T4 disease, more commonly with locoregional lymph node spread.

“Short course” RT is given without concurrent chemotherapy and is considered an alternative to conventional long-course therapy. Its use has also been studied in the T3/T4, node-positive clinical setting, but it is more commonly utilised in T3 disease without lymph node spread, or with only 1 or 2 positive nodes not geographically near to the MRF. The dose is 25 Gy in 5 fx to the pelvis, and once again can utilise either 3D planning or IMRT/VMAT. Surgery can be executed immediately after RT completion (within 1 week) or can be delayed (4–8 weeks).

Over the last few years, a more aggressive approach has been studied and is gaining clinical traction. TNT involves either short-course RT alone or long-course chemo-RT combined with a longer course of chemotherapy. An example would include short-course RT followed by 6 cycles of CAPOX or 9 cycles of FOLFOX4 chemotherapy followed by surgery. Another example would be FOLFIRNOX x 6 cycles followed by long-course chemo-RT prior to surgery. RT treatment planning is similar to that discussed above. Here, we explore the high-level evidence that has led to the above standards of care.

Prior to the mid-1980s, the standard management for rectal cancer was surgical resection alone. This changed after the publication of the Gastrointestinal Tumour Study Group trial in 1985 with an update in 1988 [57,58]. The trial randomised 227 patients with the modern equivalent of T3-4 and/or node-positive rectal cancer to 4 arms: (1) surgery alone, (2) surgery with adjuvant semustine chemotherapy, (3) surgery with adjuvant RT (up to 48 Gy), and (4) surgery with adjuvant combined chemo-RT followed by adjuvant semustine. Adjuvant chemo-RT improved 7-year local control, and disease-free and overall survival compared to all other arms. There was also a reduced rate of distant metastasis in that arm. There was increased acute toxicity in the chemo-RT arm, as would be expected. This trial established post-operative combined chemo-RT as the standard of care.

For decades, this standard persisted until the publication of the German trial by Sauer et al. in 2004 with an update in 2012 [59,60]. This trial randomised patients to pre-operative (pre-op) or post-operative (post-op) chemo-RT. The RT dose was 50.4 Gy in 28 fx in both arms (however, there was an additional 5.4 Gy boost in the post-op arm). In the pre-op arm, TME was performed at 6 weeks. Treatment compliance was much higher in the pre-op arm (90 vs. 50%). Local control was significantly improved in the pre-op arm (10.1 vs. 7.1%) as was the rate of sphincter preserving therapy (39 vs. 19%). Acute and late grade 3/4 toxicity was improved in the experimental arm. There was no statistical difference in distant recurrence, disease-free or overall survival. Neoadjuvant long-course chemo-RT has remained the standard of care in the current era.

In the early 2000s, the use of short-course RT became more prevalent. The Dutch group published a randomised study originally in 2001, with updates in 2007 and 2011 [61,62,63]. In this study, patients were randomised to either TME alone or pre-op short course RT followed by TME within a week. Ten-year local control was improved in the experimental group from 11% to 5%, with no reported difference in distant metastasis between groups. Although there was no difference seen in general overall survival, there was a benefit observed in stage III patients who had a negative circumferential resection margin.

The Trans-Tasman Radiation Oncology Group trial compared short-course RT to long-course chemo-RT in patients with T3N+ disease [64]. Surgery was performed within a week of RT completion. This seminal trial was published in 2012 and found no significant difference between the groups in local recurrence (7.5 vs. 4.4%, *p* = 0.24), distant recurrence, overall survival, or late grade 3–4 toxicity.

A Polish trial with a similar design was published prior to the Trans-Tasman trial in 2006 [65]. This study also randomised T3/4 rectal cancer patients to either short-course pre-op RT (surgery within a week) or long-course chemo-RT (surgery in 4–6 weeks). This trial also showed no differences in overall survival, local control, or late toxicity. There was also no significant difference between groups for the primary endpoint, which was the rate of sphincter preservation (61.2% vs. 58%). Note should be made that there was no standard ultrasound (U/S) or magnetic resonance imaging (MRI) mandated in this trial and not all patients received TME.

The question of when to do surgery after short-course RT is an important one. The classic Stockholm III study had a complex randomisation design [66]. There were 3 study arms: (1) short-course RT followed by TME within 7 days, (2) short-course RT followed by TME within 4–8 weeks and (3) long-course RT (with no concurrent chemotherapy) followed by TME within 4–8 weeks. Patients could be randomised to one of the 3 arms, or to one of the 2 short-course RT arms. Given that neoadjuvant long-course radiotherapy without chemotherapy is not considered to be the standard of care, we will focus our discussion on a comparison between the two short-course RT arms. There was no statistical difference found in local control between the 2 arms (2.2% vs. 2.8%). There were also no differences seen between groups for disease-free, recurrence-free, or overall survival. However, there were lower rates of post-operative (53% vs. 41%) and surgical complications (36 vs. 28%) found in the delayed TME arm.

A smaller Polish study published in 2012 also explored this important question [67]. Pach et al. randomised 154 patients to early (7–10 days) vs. delayed (4–5 weeks) surgery after short-course RT. Interestingly, there was significantly more down-staging found in the delayed RT arm (44% vs. 13%). There was no difference observed in overall survival between groups generally, but the suggestion was made that in patients with down-staging survival may be improved. There were no differences observed in sphincter-sparing surgery or local control.

There have been three phase 3 randomised controlled trials that show favorable results when comparing TNT to conventional chemo-RT. The first two trials discussed here examined short-course RT followed by several months of chemotherapy. The RAPIDO trial enrolled patients with high-risk features (T4, N2, extramural vascular invasion (EMVI), mesorectal fascia (MRF) involvement) and published results in 2020 [68]. The experimental arm was 25 Gy in 5 fx followed by either CAPOX x 6 cycles or FOLFOX4 x 9 cycles. TME was delivered within 2–4 weeks of finishing chemotherapy. The control chemo-RT arm radiotherapy dose was 50–50.4 Gy in 25–28 fx. TME was performed within 6–8 weeks of finishing RT. The primary endpoint of local or distant disease-related failure was superior in the experimental arm at 3 years (23.7 vs. 30.4%). There were also fewer metastases seen in the experimental group at 3 years (HR 0.69, CI 0.54–0.90, *p* = 0.0048). Although there was no difference seen in locoregional failure, there was a higher rate of pathologically complete responses (ypT0N0) seen in the TNT arm (28 vs. 14%). No difference in overall survival between arms was seen. Acute adverse events were higher in the TNT arm, as expected, but long-term toxicity was comparable in both arms.

The multicenter, Chinese trial STELLAR had a similar design to RAPIDO and was published earlier this year [69]. Unlike RAPIDO, however, it also included T3 and N1 patients. The TNT arm was short-course RT (25/5) followed by CAPOX chemotherapy x 4 cycles followed by TME. The control arm was standard long-course chemo-RT. The primary endpoint of disease-free survival at 3 years was 64.5 vs. 62.3% favoring the experimental arm (*p* < 0.001) and, interestingly, patients in the TNT arm also had improved overall survival (86.5 vs. 75.1%, *p* = 0.033). The ypT0N0 rate was once again found to be higher in the experimental arm (21.8 vs. 12.3%, *p* = 0.002). Acute toxicity was, unsurprisingly, higher in the TNT arm. There were no meaningful differences observed in metastasis-free survival or locoregional recurrence.

The French UNICANCER-PRODIGE 23 trial was published in 2021 [70]. This innovative trial randomised T3-4, N+ rectal cancer patients to the control (long-course chemo-RT) or long-course TNT arm. Patients in the experimental arm received 6 cycles of FOLFIRNOX chemotherapy prior to initiating long-course, concurrent chemo-RT to a dose of 50 Gy in 25 fx. TME was performed 6–8 weeks after completion of RT. The primary outcome, disease-free survival at 3 years, was significantly improved in the TNT arm (76 vs. 69%) as was metastasis-free survival (HR 0.64, CI 0.44–0.93, *p* = 0.017). ypT0N0 rate was 28 vs. 12% favoring the experimental arm. There was no difference observed in overall survival. Similar to the other TNT arms, acute toxicity was higher in the experimental arm, but similar in regard to late toxicity. There was a lower rate of grade 3 peripheral neuropathy observed in the experimental arm.

Neoadjuvant RT is considered the standard of care in locally advanced, stage II or stage III rectal cancer. Established strategies include long-course concurrent chemo-RT or short-course RT alone. However, an emerging paradigm shift is occurring towards the use of TNT as a new standard of care. Individual patient cases should be discussed amongst colleagues and at multidisciplinary tumour boards to determine which approach is optimal.

## 7. Question 6

Which patients are best suited for an organ preservation approach with clinical stage I-III rectal cancer?

### 7.1. Recommendations

- Transanal endoscopic microsurgery (TEM) is a standard of care for low-risk T1 patients when the risk of nodal involvement is <10%.
- If high-risk T1 disease that predicts lymph node metastases is found on TEM and patients cannot proceed with resection, radiation plus or minus chemotherapy should be considered [71,72,73].
- The current standard approach for all other patients, including those who achieve complete clinical response to neoadjuvant therapy, is definitive surgical resection.
- In patients who proceed with an organ preservation approach, patients and surgeons must be committed to an intensive surveillance strategy in order to detect early recurrence of cancer in a third of cases. These cases should be discussed within a multidisciplinary team [74].

### 7.2. Summary of Evidence

Radical resection for rectal cancer confers major morbidity that may include a permanent colostomy. Organ preservation using transanal excision has been performed for small lesions within 10 cm of the anal verge but with a high local recurrence of up to 30% [75,76]. The high local recurrence is variable depending on the cancer stage and the ability of the surgeon to adequately visualise excision margins through the small anal opening [77]. Technical advances in rectal endoluminal insufflation and magnification, TEM, have improved visualisation of excision margins with anticipation of improved local recurrence with sphincter preserving transanal excision [78].

Evidence is limited for organ-preserving local excision using TEM vs. gold-standard radical resection with TME [79]. There are 4 randomised trials comparing TEM vs. TME with a total of only a small number of patients [80,81,82,83]. In these trials, local recurrence for TEM was 0–9% vs. 0–2% for radical resection. Disease-free survival was 86% with TEM vs. TME 92% and overall survival of 91% vs. 93%.

Adjuvant and neoadjuvant radiation may improve local recurrence after transanal local excision [71,72,73,74,77]. However, radiation is associated with suture line dehiscence, worse anorectal function, incontinence, increased stool frequency, incomplete rectal evacuation, and low anterior resection syndrome [83].

Nevertheless, TEM is preferred by patients over radical resection on the basis of organ preservation that avoids a permanent colostomy. Further, TEM has less morbidity and pain, and can be performed as a day surgery with faster return to work and usual activities [84].

Organ-preserving local excision does not treat mesorectal lymph nodes. Mesorectal lymphadenopathy is predicted by T-stage, degree of differentiation and lymphovascular invasion, depth of submucosal invasion and tumour budding [85,86,87]. T-stage is assessed using endorectal ultrasound and MR with an accuracy of 70–90% [88,89].

Colonoscopic polypectomy is inadequate for achieving a clear submucosal or deep excision margin in most cases. Colonoscopic piecemeal excision is inadequate for assessing deep excision margin. Endoscopic submucosal dissection, ESD, with en bloc excision may provide an adequate assessment of the depth of invasion.

The current strategy for the most accurate T-stage assessment is TEM full-thickness excision of a disc of the rectal wall and peri-rectal fat without compromising the circumferential mesorectal fascia. This excisional biopsy is examined histologically for excision margins, depth of cancer invasion, degree of differentiation, and presence of lymphovascular invasion and tumour budding. If risk factors predict lymph node malignancy, the recommendation is radical resection with TME rather than adjuvant radiation.

The recommendation for organ preservation approach for early rectal cancer is T1N0 stage with low risk for metastases as assessed using TEM full thickness excisional biopsy, MR and CT. The patient and surgeon/physician must commit to close surveillance.

## Figures and Tables

**Table 1:** Attendees at the 24th Annual Western Canadian Gastrointestinal Cancer Consensus Conference.

Name	Position	Organization
Gill, Sharlene	Medical Oncologist	British Columbia Cancer Agency—Vancouver
Ahmed, Shahid	Medical Oncologist	Saskatchewan Cancer Agency
Anderson, Brady	Medical Oncologist	Western Manitoba Cancer Center
Berry, Scott	Medical Oncologist	Queen’s University
Gill, Karamjit	Medical Oncologist	British Columbia Cancer Agency—Vancouver
Iqbal, Mussawar	Medical Oncologist	Allan Blair Cancer Centre
Lim, Howard	Medical Oncologist	British Columbia Cancer Agency—Vancouver
Phang, Terry	Professor of Surgery	University of British Columbia
Sharma, Ankur	Radiation Oncologist/ Clinical Assistant Professor	Central Alberta Cancer Centre/University of Calgary Cumming School of Medicine
Solar Vasconcelos, Joao Paulo	GI Medical Oncology Fellow	British Columbia Cancer Agency—Vancouver
Tankel, Keith	Radiation Oncologist	Cross Cancer Institute
Chan, Theresa	Medical Oncologist	British Columbia Cancer Agency—Surrey
Recsky, Magdalena	Surgeon	Kelowna General Hospital
Nuk, Jennifer	Practice Leader— Genetic Counselling	BC Cancer Hereditary Cancer Program
Paul, James	Medical Oncologist	CancerCare Manitoba/University of Manitoba

**Table 2:** The Clinical Questions Addressed as Part of the 24th Annual WCGCCC, to Address Specific Aspects of Interest in Gastrointestinal Cancer.

Number	Question
**1**	What is the role of immunotherapy in deficient mismatch repair (dMMR) early-stage rectal cancer? Can a non-operative approach be considered in patients who experience a complete response?
**2**	Can circulating tumour DNA (ctDNA) be used to tailor adjuvant chemotherapy in stage II colon cancer?
**3**	What is the role of chemotherapy in combination with monoclonal antibodies (mAbs) for the first-line treatment of patients with metastatic colorectal cancer?
**4**	Which patients with newly diagnosed colorectal cancer should be referred for genetic screening?
**5**	Which patients with clinical stage II/III rectal cancer should preferably receive long-course chemoradiation over short-course radiation?
**6**	Which patients are best suited for an organ preservation approach with clinical stage I-III rectal cancer?

**Table 3:** Which patients with newly-diagnosed CRC should be referred for germline genetic screening/assessment?

□ dMMR colorectal cancer (not attributed to *MLH1* promoter methylation)
□ Irrespective of MMR status: □ Diagnosis < 50 years of age □ Multiple Lynch syndrome primary tumours any age * □ PREMM5 score ≥ 2.5% or MMRpro/MMRpredict score ≥ 5%
□ ≥10 cumulative colorectal adenomas at any age
□ ≥3 cumulative gastrointestinal hamartomatous polyps: hamartomas, ganglioneuromas, Peutz–Jeghers polyps, juvenile polyps
□ ≥5 serrated lesions proximal to the rectum (all ≥5 mm in size; with ≥2 being ≥10 mm) OR >20 throughout the large bowel, with ≥5 proximal to the rectum ^†^
□ Known pathogenic or likely pathogenic variant in a family member
□ At least one FDR with colorectal or endometrial cancer
□ 1 FDR or SDR with Lynch syndrome-related cancer * diagnosed <50 years of age
□ ≥2 FDR or SDR with a Lynch syndrome-related cancer * regardless of age
□ Pathogenic or likely pathogenic variant on tumour sequencing in relevant gene; private pay, clinical trial, research, etc.

* Colorectal, endometrial, gastric, ovarian, pancreas, urothelial, brain (usually glioblastoma), biliary tract, small intestine, sebaceous adenomas, sebaceous carcinomas, and keratoacanthomas (cancers in bold). † Serrated polyposis syndrome (SPS) often a clinical diagnosis; due to phenotypic overlap, germline genetic testing may be offered. Note: dMMR–deficient mismatch repair; MMR–mismatch repair; PREMM5, MMRPro, and MMRPredict–models to identify MMR gene mutation carriers from non-carriers; FDR–first-degree relative; SDR–second-degree relative.
