# Correction: Gill et al. Report from the 24th Annual Western Canadian Gastrointestinal Cancer Consensus Conference on Colorectal Cancer, Richmond, British Columbia, 28–29, October 2022. *Curr. Oncol.* 2023, *30*, 7964–7983

**DOI:** 10.3390/curroncol31060246

**Published:** 2024-06-03

**Authors:** Sharlene Gill, Shahid Ahmed, Brady Anderson, Scott Berry, Howard Lim, Terry Phang, Ankur Sharma, Joao Paulo Solar Vasconcelos, Karamjit Gill, Mussawar Iqbal, Keith Tankel, Theresa Chan, Magdalena Recsky, Jennifer Nuk, James Paul, Shazia Mahmood, Karen Mulder

**Affiliations:** 1 British Columbia Cancer Agency, Vancouver, BC V5Z 4E6, Canada; hlim@bccancer.bc.ca (H.L.); joao.solarvasconcelos@bccancer.bc.ca (J.P.S.V.); kgill@bccancer.bc.ca (K.G.); 2 Saskatchewan Cancer Agency, Saskatoon, SK S4W 0G3, Canada; shahid.ahmed@saskcancer.ca; 3 Western Manitoba Cancer Center, Brandon, MB R7A 5M8, Canada; banderson6@manitoba-physicians.ca; 4 Department of Oncology, Queen’s University, Kingston, ON K7L 3N6, Canada; scott.berry@kingstonhsc.ca; 5 Department of Surgery, University of British Columbia, Vancouver, BC V6T 1Z4, Canada; tphang@providencehealth.bc.ca; 6 Central Alberta Cancer Centre, School of Medicine, University of Calgary Cumming, Red Deer, AB T4N 6R2, Canada; ankur.sharma@albertahealthservices.ca; 7 Allan Blair Cancer Centre, Regina, SK S4T 7T1, Canada; mussawar.iqbal@saskcancer.ca; 8 Cross Cancer Institute, Edmonton, AB T6G 1Z2, Canada; keith.tankel@albertahealthservices.ca (K.T.); shazia.mahmood@saskcancer.ca (S.M.); karen.mulder@albertahealthservices.ca (K.M.); 9 British Columbia Cancer Agency, Surrey, BC V3V 1Z2, Canada; tchan4@bccancer.bc.ca; 10 Kelowna General Hospital, Kelowna, BC V1Y 1T2, Canada; magdalena.recsky@interiorhealth.ca; 11 British Columbia Cancer Hereditary Cancer Program, Victoria, BC V8R 6V5, Canada; jnuk@bccancer.bc.ca; 12 CancerCare Manitoba, University of Manitoba, Winnipeg, MB R3E 0V9, Canada; jpaul@cancercare.mb.ca

Karen Mulder was not included as an author in the original publication [1]. The corrected Author Contributions statement appears here. The authors state that the scientific conclusions are unaffected. This correction was approved by the Academic Editor. The original publication has also been updated.

## References

[1] Gill S., Ahmed S., Anderson B., Berry S., Lim H., Phang T., Sharma A., Solar Vasconcelos J.P., Gill K., Iqbal M. (2023). Report from the 24th Annual Western Canadian Gastrointestinal Cancer Consensus Conference on Colorectal Cancer, Richmond, British Columbia, 28–29, October 2022. Curr. Oncol..

